# Field evaluation of an automated mosquito surveillance system which classifies *Aedes* and *Culex* mosquitoes by genus and sex

**DOI:** 10.1186/s13071-024-06177-w

**Published:** 2024-03-01

**Authors:** María I. González-Pérez, Bastian Faulhaber, Carles Aranda, Mark Williams, Pancraç Villalonga, Manuel Silva, Hugo Costa Osório, Joao Encarnaçao, Sandra Talavera, Núria Busquets

**Affiliations:** 1 IRTA, Programa de Sanitat Animal, Centre de Recerca en Sanitat Animal (CReSA), Campus de la Universitat Autònoma de Barcelona (UAB), Bellaterra, Spain; 2 Unitat mixta d’Investigació IRTA-UAB en Sanitat Animal, Centre de Recerca en Sanitat Animal (CReSA), Campus de La Universitat Autònoma de Barcelona (UAB), Bellaterra, Spain; 3 Irideon S.L, Barcelona, Spain; 4 Servei de Control de Mosquits del Consell Comarcal del Baix Llobregat, El Prat de Llobregat, Spain; 5 National Institute of Health/Centre for Vectors and Infectious Diseases Research, Águas de Moura, Portugal; 6 Instituto de Saúde Ambiental, Faculdade de Medicina, Universidade de Lisboa, Lisbon, Portugal

**Keywords:** *Aedes*, Automated classification, *Culex*, Field study, Machine learning, Mosquito surveillance, Optical sensor

## Abstract

**Background:**

Mosquito-borne diseases are a major concern for public and veterinary health authorities, highlighting the importance of effective vector surveillance and control programs. Traditional surveillance methods are labor-intensive and do not provide high temporal resolution, which may hinder a full assessment of the risk of mosquito-borne pathogen transmission. Emerging technologies for automated remote mosquito monitoring have the potential to address these limitations; however, few studies have tested the performance of such systems in the field.

**Methods:**

In the present work, an optical sensor coupled to the entrance of a standard mosquito suction trap was used to record 14,067 mosquito flights of *Aedes* and *Culex* genera at four temperature regimes in the laboratory, and the resulting dataset was used to train a machine learning (ML) model. The trap, sensor, and ML model, which form the core of an automated mosquito surveillance system, were tested in the field for two classification purposes: to discriminate *Aedes* and *Culex* mosquitoes from other insects that enter the trap and to classify the target mosquitoes by genus and sex. The field performance of the system was assessed using balanced accuracy and regression metrics by comparing the classifications made by the system with those made by the manual inspection of the trap.

**Results:**

The field system discriminated the target mosquitoes (*Aedes* and *Culex* genera) with a balanced accuracy of 95.5% and classified the genus and sex of those mosquitoes with a balanced accuracy of 88.8%. An analysis of the daily and seasonal temporal dynamics of *Aedes* and *Culex* mosquito populations was also performed using the time-stamped classifications from the system.

**Conclusions:**

This study reports results for automated mosquito genus and sex classification using an optical sensor coupled to a mosquito trap in the field with highly balanced accuracy. The compatibility of the sensor with commercial mosquito traps enables the sensor to be integrated into conventional mosquito surveillance methods to provide accurate automatic monitoring with high temporal resolution of *Aedes* and *Culex* mosquitoes, two of the most concerning genera in terms of arbovirus transmission.

**Graphical Abstract:**

**Figure:**
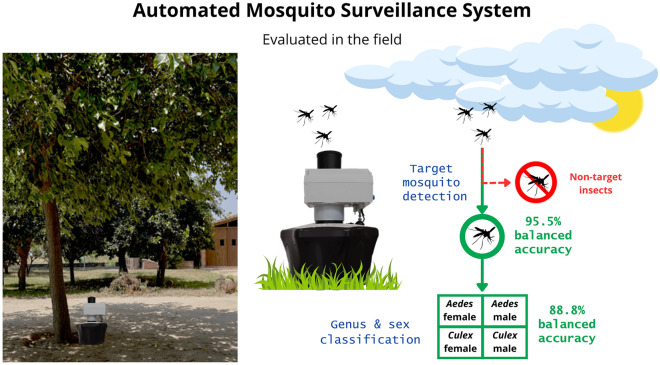

**Supplementary Information:**

The online version contains supplementary material available at 10.1186/s13071-024-06177-w.

## Background

Mosquitoes (Diptera: Culicidae) act as vectors of several pathogens such as malaria parasite, dengue (DENV), Zika (ZIKV), yellow fever (YFV), chikungunya (CHIKV), and West Nile (WNV) viruses that cause diseases which result in hundreds of thousands of human deaths per year worldwide, primarily in tropical countries of Africa, East and Southeast Asia, and South America [1]. In Europe, autochthonous vector species such as *Culex pipiens* and invasive vector species such as *Aedes albopictus* are responsible for the transmission of endemic (e.g. WNV, Usutu, Sindbis, Tahyna, and Batai viruses, lymphatic filariasis, and avian malaria) and imported (CHIKV, DENV, and ZIKV) pathogens, respectively, and pose a threat to public and veterinary health in the continent [2]. To mitigate the impact of mosquito-borne diseases (MBD), surveillance programs for both native and invasive species are used by public health organizations worldwide to monitor trends in vector populations and to assess the effectiveness of control programs [3, 4]. The availability of high-quality surveillance data is essential for these tasks and to model the risk of MBD [5]. 

Traditional entomological methods for mosquito monitoring generally entail the use of physical traps, which primarily target adult mosquitoes as a proxy for pathogen transmission risk [6]. These methods are very costly in terms of the human resources involved in the tasks of sample collection in the field, taxonomical identification of the samples, and data processing. Furthermore, the time lag between the time of capture and the analysis of the samples and processing of the results may hinder a full understanding of the real-time dynamics of mosquito populations. This delay can limit the proper assessment of disease transmission risk and the timely application of control measures. Consequently, the application of new technologies, including machine learning (ML), to the automated and remote real-time characterization of mosquito populations may have a positive impact on the state of the art in entomological surveillance [7, 8].

Over recent years, there has been an increasing number of studies aimed at taxonomically classifying mosquitoes and other attributes of mosquito biology using either acoustic [9–12] or optical sensors [13–19], which take advantage of insect bioacoustic properties. The study of these properties, especially the mosquito flight tone or wing beat frequency, has been used for mosquito characterization and classification purposes since the 1940s [20, 21]. However, the existence of overlapping frequency distributions among different mosquito species [14, 22] led to the exploration of other predictor variables such as spectrograms, power spectral density, Mel frequency cepstral coefficients, and optical depolarization ratio, which provide better classification results [15, 17, 19]. In addition to the choice of features, the choice of ML algorithm and its configuration parameters has been shown to contribute to the overall classification accuracy [15, 17, 19].

Despite the growth of research in automated remote mosquito surveillance [8], few published papers describe the evaluation of solutions in the field [10, 23, 24]. Technical constraints such as interference from ambient noise in the case of acoustic sensors [22], presence of heavy rain during the sampling period [25], proportion of mosquitoes relative to other flying insects in the capture [23], capture efficiency of the sampling devices [24], and ambient environmental temperature, which is known to affect mosquito flight tone [26], may limit the usage of these systems for field monitoring of mosquito populations. The only example of a commercial mosquito sensor with reported results is the BG-Counter (Biogents, Regensberg, Germany) [25], which is claimed to distinguish mosquitoes from other insect species and whose performance was shown to have a high rate of misclassifications when the proportion of non-mosquitoes was significant [23].

In this contribution, we present the results of a field study of an automated mosquito surveillance system in which an optical sensor coupled to the entrance of a standard mosquito suction trap automatically differentiated target mosquitoes (*Aedes* and *Culex*) from other insects that enter the trap and identified the genus and sex of these target mosquitoes. We previously reported high levels of accuracy for genus and sex classification of *Aedes* and *Culex* mosquitoes in the laboratory using the same technology [19]. In the current study, a new ML dataset was built with 14,067 mosquito flights in the laboratory, corresponding to a wider range of larval density and ambient temperature conditions to cover the morphological variability and ambient temperature range of the target genera in nature. A new ML model was trained using this dataset. The sensor and trap were deployed and assessed in the field during periods of mosquito activity at two different locations in a Mediterranean climate area with a predominance of *Cx. pipiens* and *Ae. albopictus*, potential vectors of imported and endemic arboviruses.

## Methods

### Optical sensor

**Fig. 1:**
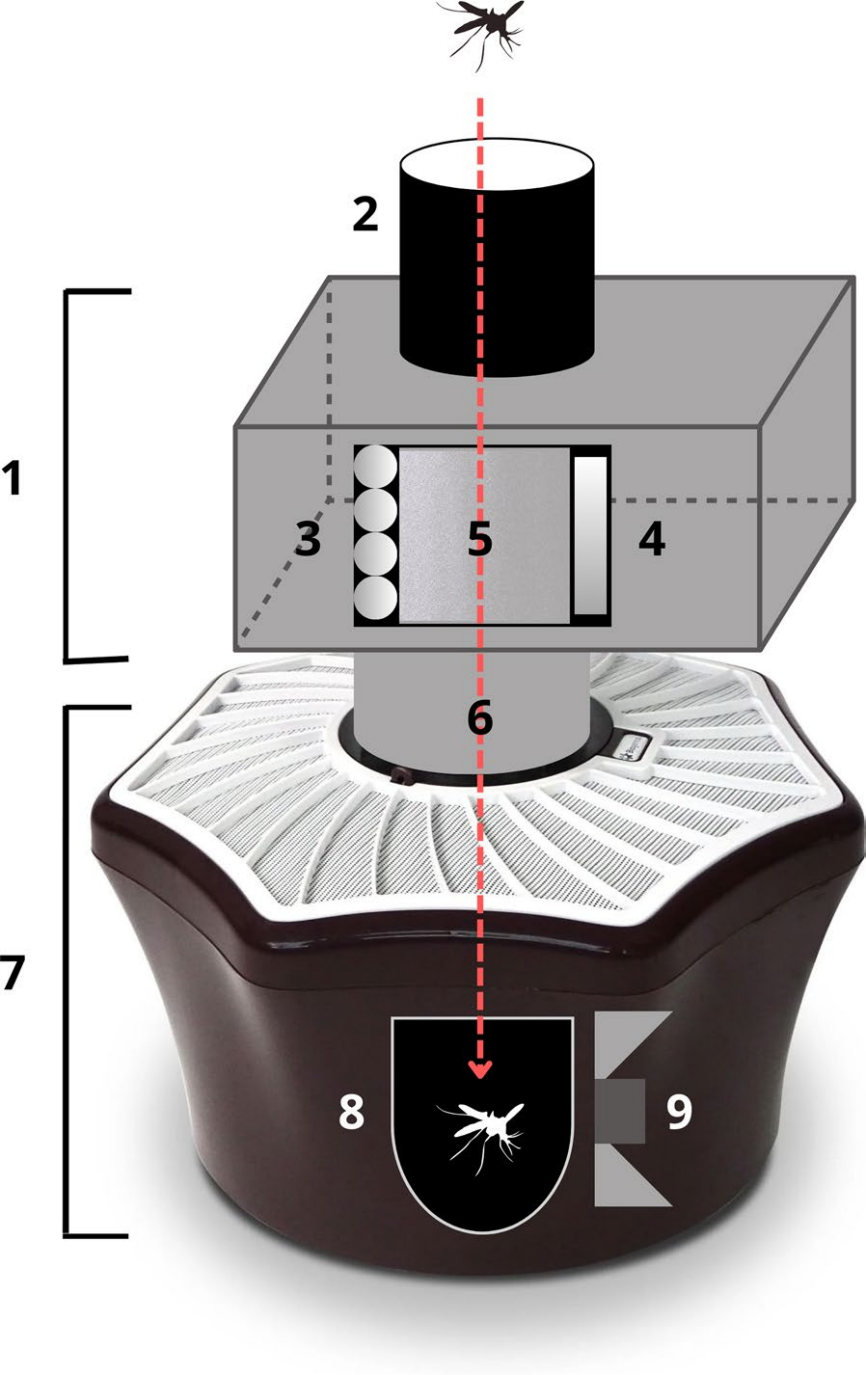
Components of the optical sensor and trap system: (1) exterior of the sensor unit; (2) inlet tube; (3) optical emitter; (4) optical receiver; (5) field of view of the sensor; (6) outlet tube; (7) mosquito suction trap; (8) catch bag; (9) fan

The optical sensor (Irideon, Barcelona, Spain) comprises a light gray waterproof enclosure (width 25.5 × diameter 18 × height 13 cm) with a black inlet tube of 10-cm diameter at the top of the unit and a light gray outlet tube on the underside. The sensor contains an optical emitter, comprising a rectangular array of 940-nm wavelength light emitting diodes (LEDs), which together emit a collimated beam (width 10.5 × height 7 cm) of near-infrared light towards an optical receiver formed by a corresponding array of photodiodes. The emitter and receiver face each other through a transparent circular tube of 10.5 cm diameter which traverses the enclosure from top to bottom to create a sensing zone with a volume of 600 cm^3^. The sensor was placed on the entrance of a BG-Mosquitaire mosquito trap (Biogents, Regensburg, Germany). The trap contains a suction fan, removable catch bag, and flap valve, which automatically opens when the fan is powered. The suction fan creates a downward flow of air through the inlet tube of the sensor and into the trap. When an insect flies close to opening of the inlet tube, it is likely to be sucked into the tube, down through the sensing zone, and through the flap valve and into the catch bag. As the flying insect passes through the sensing zone, it casts a fast-changing shadow upon the optical receiver because of the modulation of the light beam by the wing flap of the insect, and this signal is recorded by the sensor. Two cables exit the sensor: one is connected to a 12-VDC power supply, such as the supply included with the BG Mosquitaire trap, and the other is connected to the trap to power the fan. A diagram which illustrates the operation of the sensor and trap is shown in Fig. 1.

Two variants of the sensor were used in the present work: a laboratory version, which was used to record mosquito flights in the laboratory to build the ML dataset, and a field version, which was used to record mosquito flights in the field for automated mosquito classification using the ML model previously trained with the laboratory data. The two variants differ only by their method of data communication, which was via USB to a laptop computer for laboratory use, or wireless communication via the mobile phone network to a server for field use. Further details about the sensor were reported in our previous work [19].

### Mosquito-rearing conditions for the creation of the dataset

Two populations of *Ae. albopictus* and one population of *Cx. pipiens* were reared in the laboratory from immature stages (eggs and larvae respectively) collected in the field: *Ae. albopictus*, population of Rubí (2020), Barcelona, Spain (41.50674, 2.00778); *Ae. albopictus*, population of Vilamoura (2020–2022), Algarve, Portugal (37.08546, 8.11929); and *Cx. pipiens*, population of Bellaterra (2020, 2022), Cerdanyola del Vallés, Barcelona, Spain (41.49903, 2.10872). The mosquito strains obtained in Barcelona were reared in the insectarium facilities of IRTA-CReSA (Campus of the Autonomous University of Barcelona, Cerdanyola del Vallès, Barcelona, Spain). The *Ae. albopictus* strain obtained in Portugal was reared in the insectarium facilities of CEVDI/INSA (Águas de Moura, Setúbal, Portugal).

Larvae were maintained in plastic trays with two larval density regimes (50 and 250 larvae/tray) in 750 ml of dechlorinated tap water, renewed three times per week, and fed with fish food pellets (Goldfish Sticks-TETRA, Melle, Germany). Pupae were placed in plastic cups inside insect-rearing cages with dimensions of 30 × 30 × 30 cm (BugDorm-1 Insect Rearing Cage, MegaView Science, Talchung, Taiwan). Adults were fed with 10% sucrose solution ad libitum, which was removed 24 h before the flight assays of the females to increase their appetite, host-seeking activity, and likelihood of entering the trap. All females used in the experiment were nulliparous, and their age ranged from 2 to 16 days. The age of the males ranged from 2 to 9 days.

Each development stage of the mosquito life cycle took place inside a climatic chamber at controlled environmental conditions of: 28 °C temperature, 80% relative humidity, and a light:dark photoperiod of 12:12 h for *Ae. albopictus* and 11:11 h (plus 1 h of dusk and 1 h of dawn) for *Cx. pipiens*. All mosquito colonies were maintained until a maximum of 15 generations to minimize any changes to flight characteristics due to the adaptation of wild populations to prolonged confinement.

### Flight assays in the laboratory and training of the machine learning model

The sensor and trap were placed in an insect cage (BugDorm-4S4590 width 47.5 × diameter 47.5 × height 93.0 cm, MegaView Science, Talchung, Taiwan) inside a climatic chamber. The trap was fitted with a sachet of BG-Sweetscent chemical attractant (Biogents, Regensberg, Germany) to attract mosquitoes towards the sensor and trap. Flight assays were performed at different temperatures to cover the range of temperature at which the assayed mosquito species are known to have flight activity: ~ 15–35 °C for *Ae. albopictus* [27] and ~ 15–30 °C for *Cx. pipiens* [28].

Before the flight assays, mosquitoes were anesthetized using carbon dioxide and separated in small cardboard boxes sorted by genus (*Aedes* or *Culex*) and sex (female or male). All mosquitoes were held in the climatic chamber at the designated ambient temperature for 24 h prior to the start of the assay to acclimatize them. They were then released into the insect cage containing the sensor in batches of 25 individuals every 15 min. *Aedes albopictus* and *Cx. pipiens* were assayed at 18 °C, 23 °C, and 28 °C in the facilities of IRTA-CReSA (in a climatic chamber: CCK-0/5930 m, Dycometal, Barcelona, Spain). *Aedes albopictus* was also assayed at 33 °C in the facilities of CEVDI/INSA (in a climatic chamber: FITOCLIMA S600, Aralab, Rio de Mouro, Portugal). They were released 30 cm from the entrance of the sensor to ensure that they could fly freely before being sucked into the sensor and to minimize the possibility of multiple insects passing through the sensor at the same time. Mosquitoes that did not enter the trap during the assay were removed from the insect cage with an electronic entomological aspirator (IA-INSECT02USB, Infoagro Systems, Madrid, Spain). After each flight assay, the catch bag was collected, and the specimens inside were frozen and then counted.

After each laboratory assay, the recordings were downloaded from the sensor to a laptop computer and then processed using a Python script to produce playable and viewable audio files.

During data cleaning, each recording was examined manually, and those considered to be invalid were excluded from the dataset, e.g. recordings containing double flights or those where the mosquito was deemed to have hit the wall of the flight tube inside the sensor. A machine learning model was generated using the methodology described in our previous work [19]. The gradient boosting algorithm using the XGBoost library [29] was trained with fourfold cross-validation on the extracted spectrograms of a balanced sub-dataset. A test set was previously separated from the dataset to evaluate the trained model on unused data.

### Field trial of the automated mosquito surveillance system

**Fig. 2:**
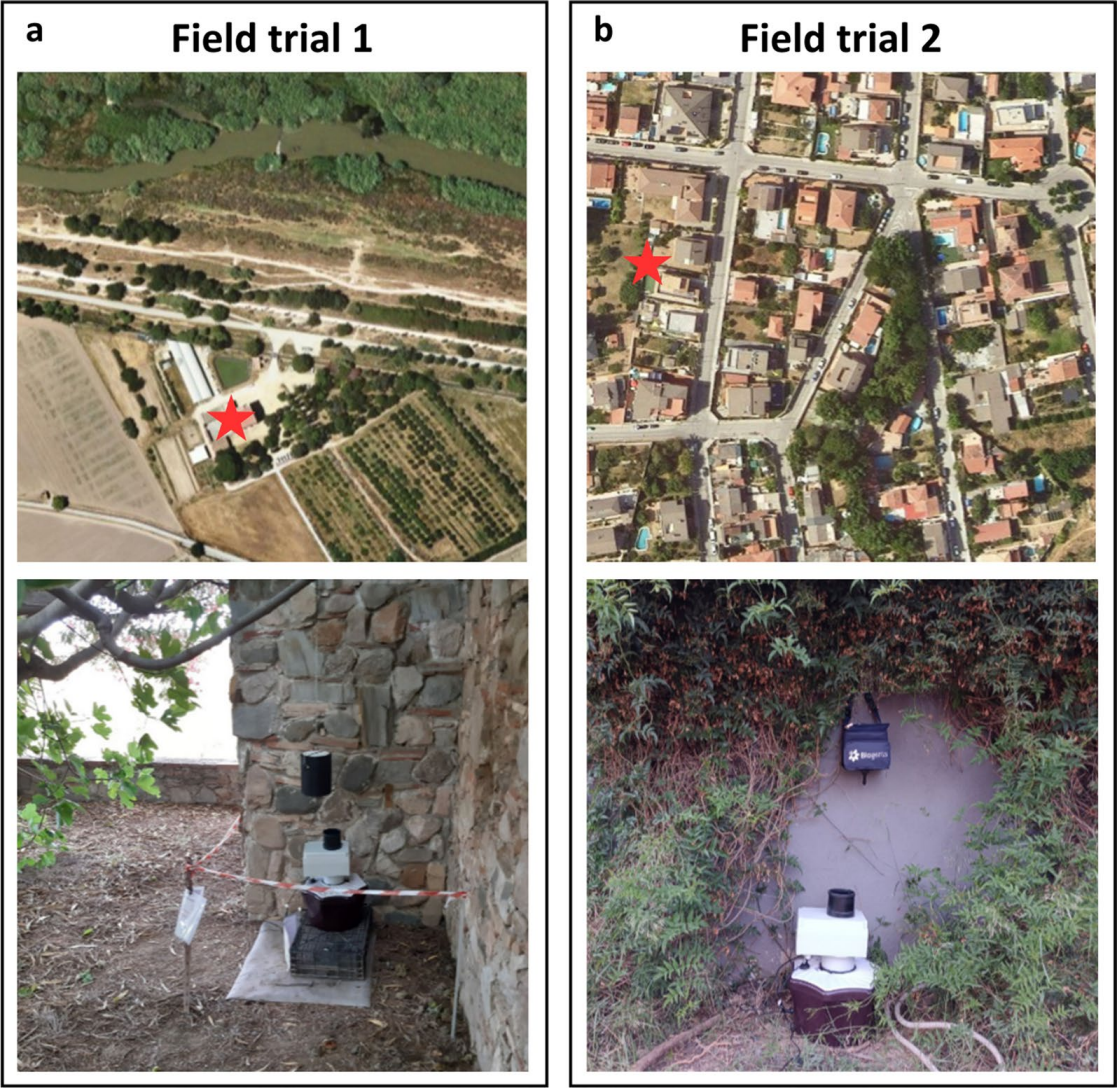
**a** Field trial 1: The sensor and trap deployed near *Can Comas*, a nineteenth century country house located in the Baix Llobregat Agrarian Park in the municipality of El Prat de Llobregat, Barcelona, Spain (41.341286, 2.078259). The park is a protected natural and rural space located in the alluvial plains of the delta and the lower basin of the Llobregat River. Land use in the area includes rainfed and irrigated agricultural crops (mainly fruit and vegetables), livestock (primarily sheep), and the Barcelona-El Prat International Airport. **b** Field trial 2: The sensor and trap deployed in the backyard of a private house in a residential area of Rubí, Barcelona, Spain (41.472816, 2.032258). The area is a typical peri-urban area, comprising detached houses with a garden or small sparsely planted orchard, some green areas with playgrounds and sport zones, roads, and services such as petrol stations and supermarkets. The neighborhood is bounded by two creeks which are tributaries to the Llobregat River

The sensor and trap were deployed in the municipalities of El Prat de Llobregat in 2021 (field trial 1) and Rubí in 2022 (field trial 2) in the province of Barcelona (Catalunya, Spain). These locations have a Mediterranean climate, typified by hot dry summers, mild rainy winters, and variable temperatures in autumn and spring. The specific location of the sensor and trap at each site (Fig. 2) was selected to provide shade, nearby vegetation, shelter from rain and wind, and access to electrical power in a place where mosquitoes were known to be present.

The field trials were performed in the months of peak mosquito activity. Field trial 1 ran from July to October 2021 and used collection cycles (the time between catch bag placement and sample collection) of 24 h. Field trial 2 ran from June to September 2022 and used collection cycles of 24 to 72 h. In each trial, dry ice was used as a source of carbon dioxide to attract mosquitoes to the trap. Samples were frozen at − 20 °C shortly after collection and inspected by a trained entomologist to taxonomically classify and count the content.

The flying insects that entered the trap were automatically detected and recorded by the sensor. Each recording included the sensor GPS coordinates, date and time (time stamp), measured ambient temperature, and relative humidity at the time of capture. The field sensor sent batches of sensor recordings via the mobile phone network to the server every 30 min. Each new field recording was automatically classified as either a target mosquito, in which case the classification model for genus and sex was applied, or a non-target. The method was based on the calculation of means and a covariance matrix for the laboratory dataset, which were used to generate a probability density function. The values from each field recording were passed to the probability density function, which returns the probability of these values occurring, and this probability was compared to a pre-defined threshold value. If the calculated probability was greater than or equal to the threshold value, then the recording was classified as a mosquito; otherwise, it was classified as a non-target. The threshold value was established based on the laboratory data and was then fine-tuned to maximize the target detection accuracy of the sensor, compared to the manual mosquito counts, using field data from previous unpublished trials. The classification results, with the associated capture time stamps and environmental data, were downloaded from the server as.csv files and used for the analysis in this work.

### Data analysis of sensor classification in the field

Two main functions of the automated mosquito surveillance system were assessed: (i) target mosquito detection, i.e. the ability of the system to discriminate *Aedes* and *Culex* target mosquitoes from non-target insects which also enter the trap, and (ii) the ability of the system to correctly classify mosquito genus and sex, i.e. to classify the *Aedes* female, *Aedes* male, *Culex* female, and *Culex* male classes.

The relationship between the sensor count (mosquitoes counted by the sensor) and manual count (mosquitoes counted by manual inspection) was assessed by correlation analysis and linear regression analysis and was visualized using a time series plot and a scatter plot of manual count versus sensor count per collection cycle. The Pearson correlation coefficient (*r*) and *P*-value for significance were obtained to analyze how both variables were related. Regression coefficients, i.e. the *R*^2^ coefficient of determination and the linear slope and intercept were calculated to indicate how well the regression predictions based on sensor count approximated the manual count. A regression slope of greater/less than one would indicate that overall, sensor counts were greater/lower than the manual counts.

A further evaluation metric used in this work was balanced accuracy (BA). This metric was determined by using the number of true positives (TP), true negatives (TN), false positives (FP), and false negatives (FN) using the manual classification as the reference. TP and TN are the numbers of positive and negative cases respectively that the system classified correctly. FP is the number of negative cases that the system incorrectly classified as positive, and FN is the number of positive cases that the system incorrectly classified as negative. To calculate TP, TN, FP, and FN for a particular class, this class was defined as the positive class and the other class(es) were defined as the negatives. For the positive class, TP equals the minimum common value of the sensor and manual count. If the sensor count was greater than the manual count, then the difference was taken as FP; otherwise, FP equaled zero. If the sensor count was less than the manual count, then the difference was taken as FN. TN is calculated by subtracting FP from the manual counts for the negatives. BA gives equal weighting to the proportion of positives and negatives that are correctly classified, and it is appropriate when classes are imbalanced [30], as is the case for the field captures in this work. The equation for BA is ${\text{BA}}=\frac{{\text{Se}}+{\text{Sp}}}{2}$ (where Se refers to sensitivity and Sp to specificity). Sensitivity, also known as true-positive rate or recall, indicates the proportion of positives that are correctly classified by the system (${\text{Se}}=\frac{{\text{TP}}}{{\text{TP}}+{\text{FN}}}$). Specificity, also known as true-negative rate, indicates the proportion of negatives that are correctly classified by the system (${\text{Sp}}=\frac{{\text{TN}}}{{\text{TN}}+{\text{FP}}}$).

The daily and seasonal temporal dynamics of *Aedes* and *Culex* mosquitoes were also analyzed by descriptive statistics using the time-stamped classification results in which the sensor genus counts per hour were averaged for each month over the length of each trial.

## Results

### Performance of the machine learning model using the laboratory data

A total of 15,208 mosquito flights were recorded in the laboratory, of which 7.5% were rejected during data cleaning to yield 14,067 valid flights. The valid flights were randomly under-sampled to obtain a balanced dataset from which 1000 flights were set aside as the test set. The trained ML model achieved an average balanced accuracy of 93.9% for the classification of *Aedes* female, *Aedes* male, *Culex* female, and *Culex* male flights in the test set. The BA results per class were: 91.0% for *Aedes* female, 93.4% for *Aedes* male, 96.7% for *Culex* female and 94.4% for *Culex* male. A confusion matrix of these classification results is shown in Additional file 1: Table S1. The same ML model was then used to classify the recordings from the sensor in the field.

### Manual classification of the field samples

A total of 53 samples (catch bags) were collected from the traps and underwent manual inspection: 32 in field trial 1 and 21 in field trial 2. Of these, seven showed signs of significant mosquito depredation and/or degradation and were excluded from the analysis because of the impact it would have on the manual count. A further two samples were also excluded because of mobile network connectivity issues during those collection cycles. Therefore, a total of 44 samples were inspected and used in the analysis: 29 from field trial 1 and 15 from field trial 2.

In total, 3634 mosquitoes were classified manually (1665 in field trial 1 and 1969 in field trial 2) comprising the following species in decreasing order of number: *Cx. pipiens*, *Ae. albopictus*, *Culiseta longiareolata, Aedes caspius* and *Coquillettidia richiardii* (Table 1). *Culex pipiens*, *Ae. albopictus* and *Cs. longiareolata* were found in both trials, while *Ae. caspius* was only found in field trial 1 during September and early October. Only one specimen of *Cq. richardii* was found, in field trial 1.

**Table 1:** Total number of mosquitoes and other insects by manual inspection of the samples in each field trial

Sample composition				Field trial 1	Field trial 2
Mosquitoes	Target	*Culex*	*Culex pipiens*	1261	1387
		*Aedes*	*Aedes albopictus*	270	543
			*Aedes caspius*	39	0
	Non-target	Other genus	*Culiseta longiareolata*	94	39
			*Coquilletidia richiardii*	1	0
Other insects	Non-target	–	Non-culicidae insects	3188	2125

The mean proportion of target mosquitoes (*Aedes* and *Culex*) compared to total insects in the samples was 32.4% in field trial 1 and 47.1% in field trial 2 with the non-target group mostly comprising Phlebotominae, Chironomidae, and a wide variety of small dipterians. The proportions of each genus and sex class within the target mosquitoes, from highest to lowest, were: *Culex* female (76.6% in field trial 1 and 67.1% in field trial 2), *Aedes* female (15.0% in field trial 1 and 18.9% in field trial 2), *Aedes* male (4.6% in field trial 1 and 9.2% in field trial 2), and *Culex* male (3.8% in field trial 1 and 4.8% in field trial 2).

### Automated target mosquito detection in the field

**Fig. 3:**
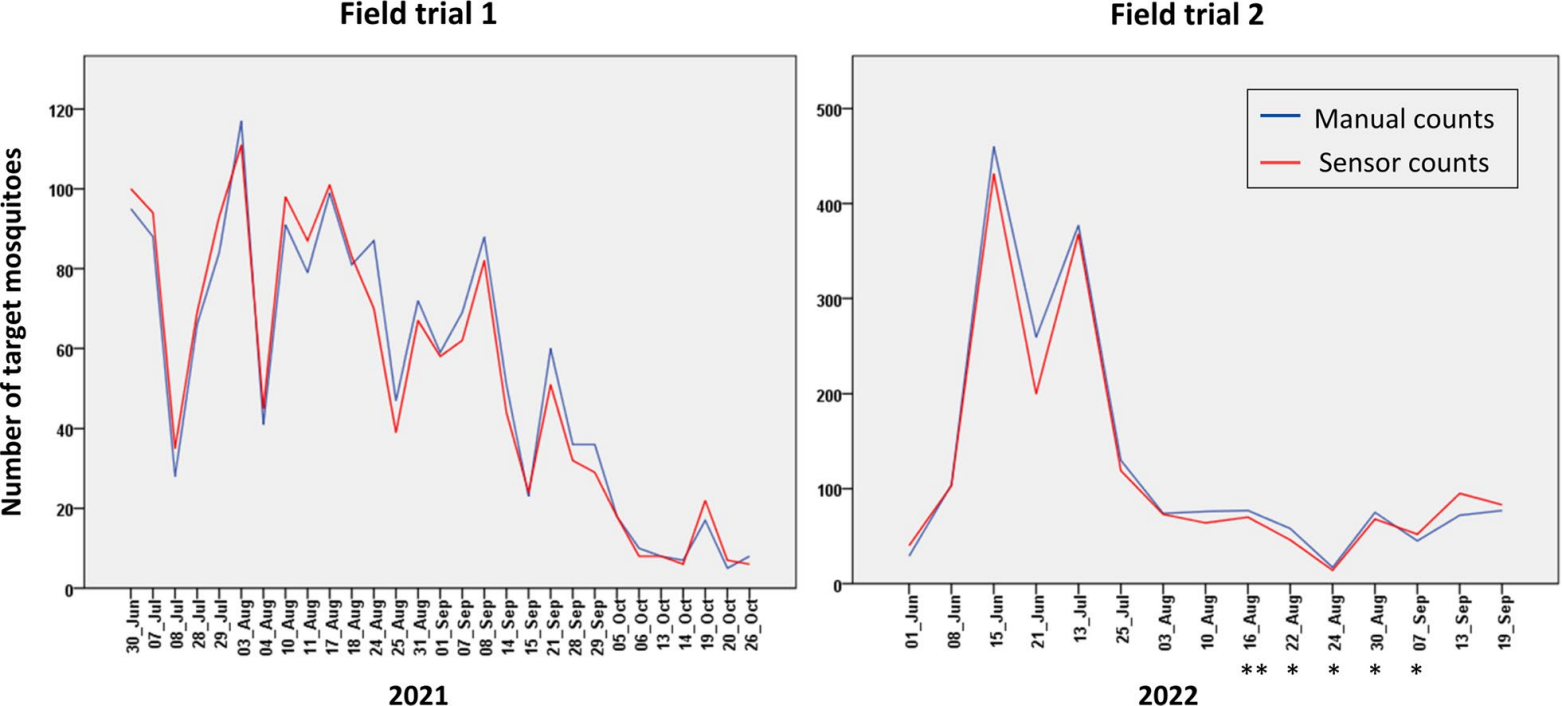
Time series plots representing the number of target mosquitoes (sensor count and manual count) per collection cycle for each field trial. The x-axis indicates the start date of each collection cycle. Collection cycles lasted 24 h in field trial 1 and 48 h in field trial 2 except those marked with *(= 24 h) or **(= 72 h)

**Fig. 4:**
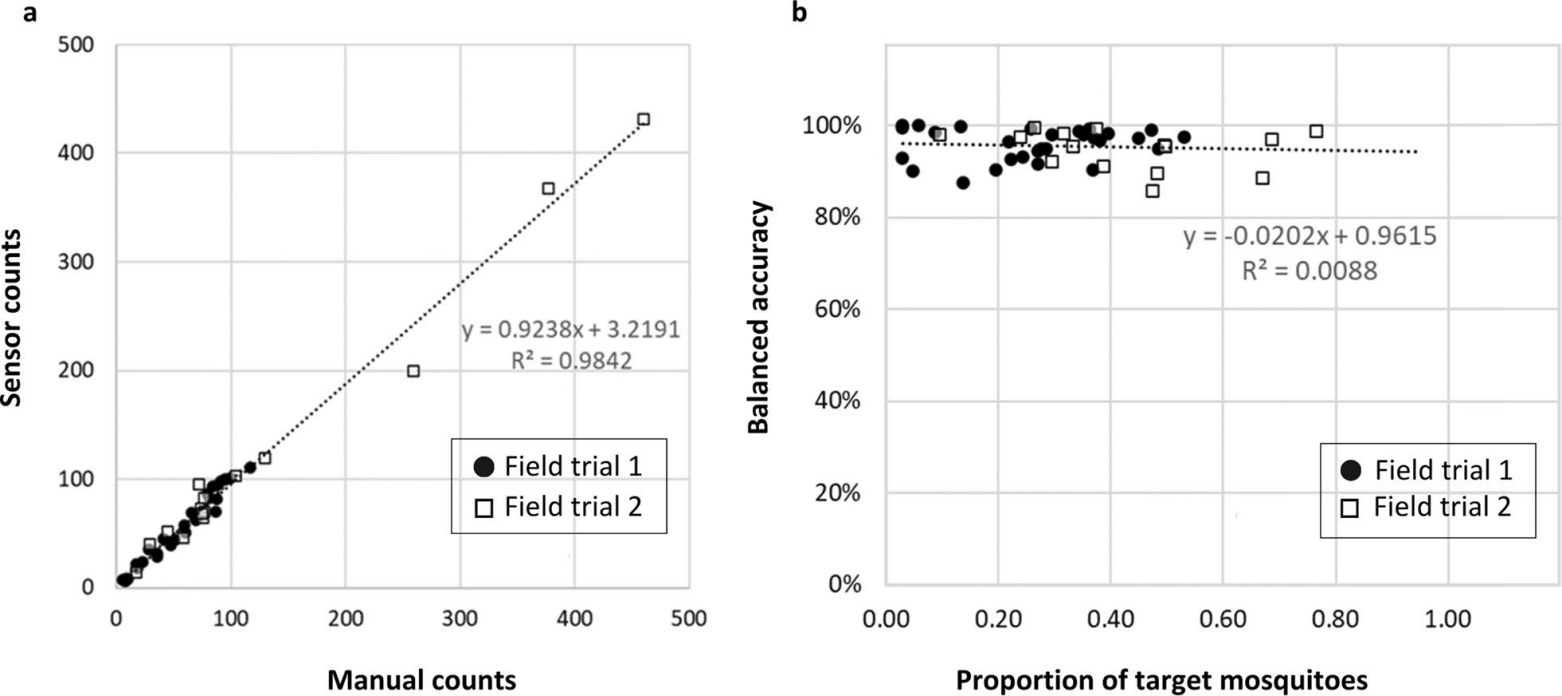
**a** Scatter plot and linear regression of sensor count versus manual count for target mosquito detection per collection cycle showing the regression line equation (slope and *y*-intercept) and coefficient of determination, *R*^2^. **b** Scatter plot and linear regression of balanced accuracy of target mosquito detection per collection cycle versus the proportion of target mosquitoes in the catch showing the regression line equation (slope and y-intercept) and coefficient of determination, *R*^2^

There was a strong positive correlation between the number of target mosquitoes counted by the sensor and by manual inspection in both field trials (*r* = 0.983, *P*-value = 0.000 in field trial 1 and *r* = 0.915, *P*-value = 0.000 in field trial 2). This good agreement between manual and sensor counts is shown in Fig. 3, even when the manual count changed significantly from one collection cycle to the next because of natural conditions. Linear regression analysis indicated a good fit of the linear regression line to manual count versus sensor count (*R*^2^ = 0.984, *P*-value = 0.000), as shown in Fig. 4a. The linear regression equation (*y* = 0.924*x* + 3.219) indicated that sensor count was typically 7.6% lower than the manual count. The manual and sensor counts for target mosquito detection are given in Additional file 1: Table S2.

The average BA for target mosquito detection per collection cycle was 95.9% in field trial 1 and 94.8% in field trial 2. Since the distribution of BA was skewed towards high values, the median, interquartile range (IQR), and first and third quartiles (Q1, Q3) are given, in Table 2. The correlation between the BA of target mosquito detection and the proportion of target mosquitoes in each collection cycle was not significant (*r* = 0.009, *P*-value = 0.956); this is also apparent in Fig. 4b, i.e. the BA of target detection was not dependent on the proportion of target mosquitoes in the samples. Furthermore, the correlation between the BA of target mosquito detection and the duration of each collection cycle (24 h in field trial 1 and 24–72 h in field trial 2) was not significant (*r* = 0.033, *P*-value = 0.834).

**Table 2:** Overall balanced accuracy results for target mosquito detection and for genus and sex classification for each field trial and for both field trials combined

	Target mosquito detection			Genus and sex classification		
	Field trial 1 (%)	Field trial 2 (%)	Field trial 1 and 2 combined (%)	Field trial 1 (%)	Field trial 2 (%)	Field trial 1 and 2 combined (%)
Average	95.9	94.8	95.5	88.0	90.5	88.8
Median	97.1	95.8	96.7	89.4	90.6	90.0
IQR	5.7	6.4	5.9	10.3	5.4	7.9
Q1	93.1	91.6	92.8	84.1	87.9	85.7
Q3	98.8	98.1	98.6	94.3	93.3	93.6

### Automated mosquito genus and sex classification in the field

**Fig. 5:**
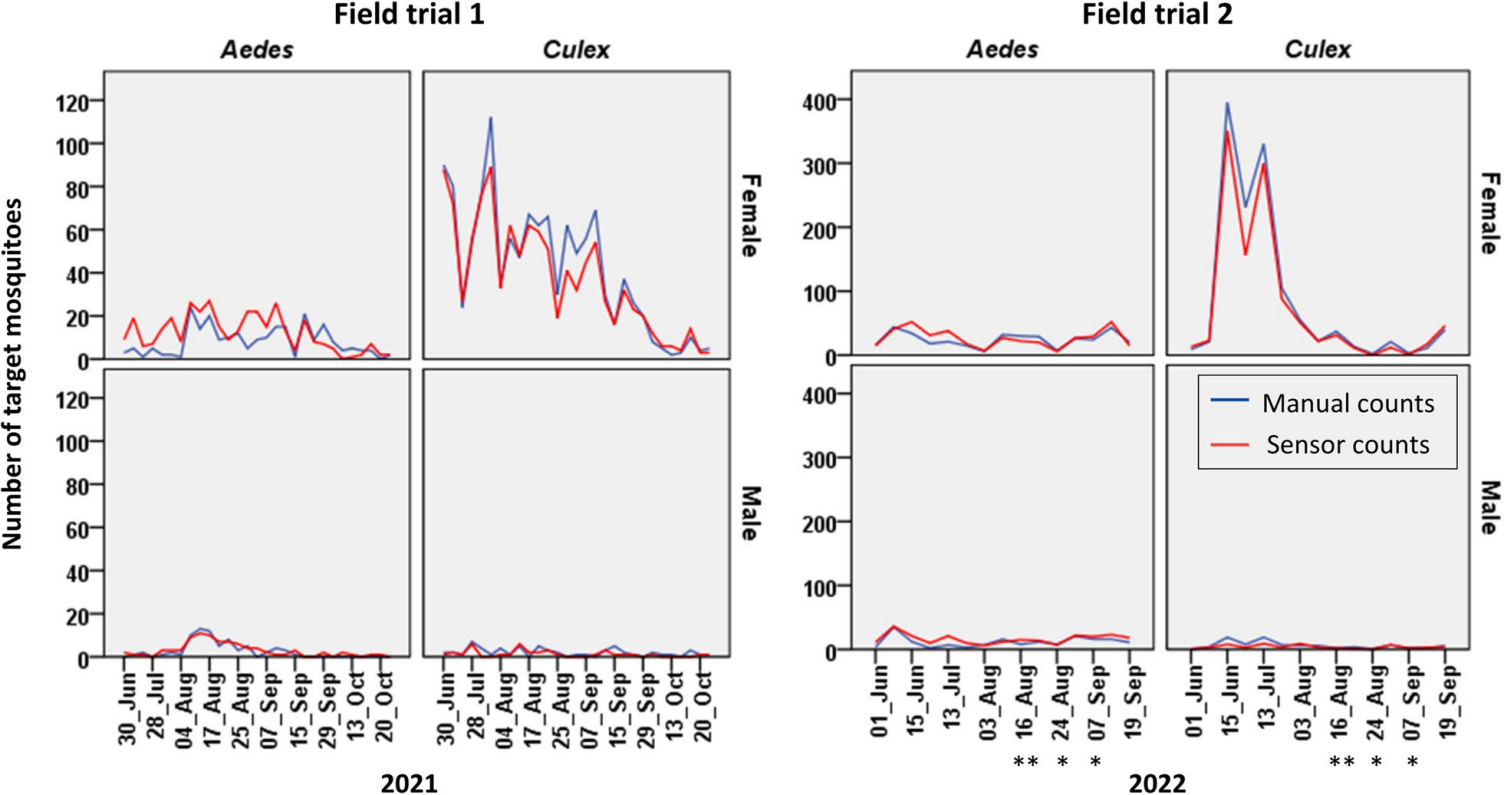
Time series plots showing the number of target mosquitoes (sensor count and manual count) for each genus and sex class per collection cycle for each field trial. The *x*-axis indicates the start date of each collection cycle. Collection cycles lasted 24 h in field trial 1 and 48 h in field trial 2 except those marked with *(= 24 h) or **(= 72 h)

**Fig. 6:**
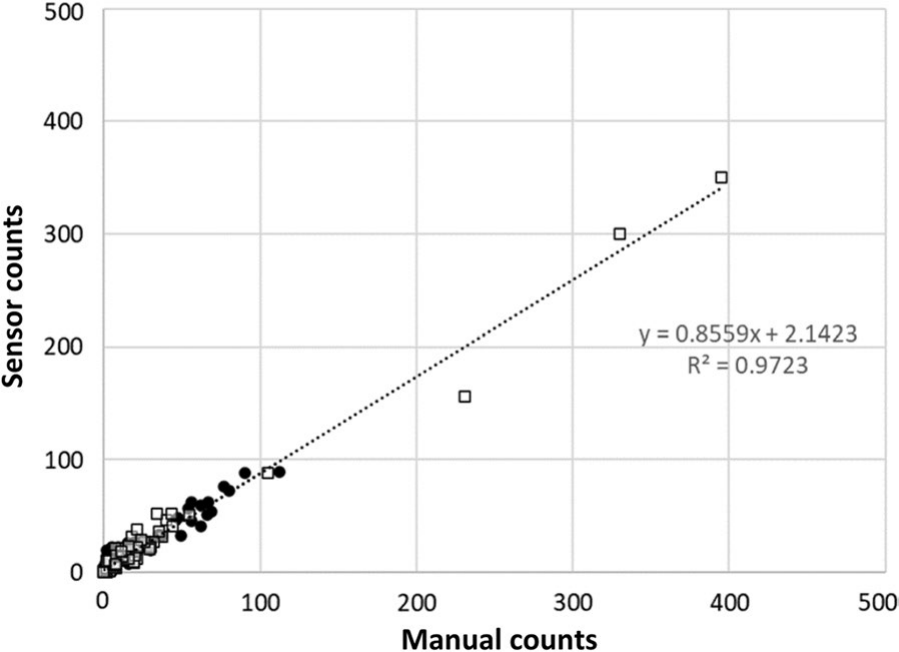
Scatter plot and linear regression of manual count versus sensor count for genus and sex detection per collection cycle showing the regression line equation (slope and y-intercept) and coefficient of determination, *R*^2^

There was a strong positive correlation (*r* = 0.846, *P*-value = 0.000 in field trial 1 and *r* = 0.903, *P*-value = 0.000 in field trial 2) between sensor counts and manual counts for the four mosquito classes (*Aedes* female, *Aedes* male, *Culex* female, and *Culex* male) in both field trials; this agreement is shown in Fig. 5. Linear regression analysis indicated a good fit of the linear regression line to the data points of manual counts versus sensor counts per collection cycle (*R*^2^ = 0.972, *P*-value = 0.000) as shown in Fig. 6. The regression equation (*y* = 0.856*x* + 2.142) indicates that the sensor count for genus and sex was typically 14.4% lower than the manual count. The manual and sensor counts for genus and sex classification are given in Additional file 1: Table S2.

The BAs, calculated over the collection cycles, for each genus and sex class were calculated for both field trials. In field trial 1, the BA results per class were: 88.8% for *Aedes* female, 93.7% for *Aedes* male, 88.9% for *Culex* female, and 80.5% for *Culex* male. In field trial 2, the BA results were: 93.3% for *Aedes* female, 95.0% for *Aedes* male, 87.8% for *Culex* female, and 85.7% for *Culex* male. The average BA of the four genus and sex classes was 88.0% in field trial 1 and 90.5% in field trial 2. Since the distribution of BA was skewed towards high values, the median, IQR, Q1, and Q3 are given, in Table 2. The BA for genus and sex was not correlated with the proportion of target mosquitoes in the samples (*r* = 0.153, *P*-value = 0.321) or to the proportion of *Aedes* (*r *= 0.262, *P*-value = 0.086), *Culex* (*r* = 0.146, *P*-value = 0.345), females (*r* = − 0.048, *P*-value = 0.756), or males (*r* = 0.037, *P*-value = 0.810) among the target mosquitoes.

### Time resolution of the automated mosquito surveillance system

**Fig. 7:**
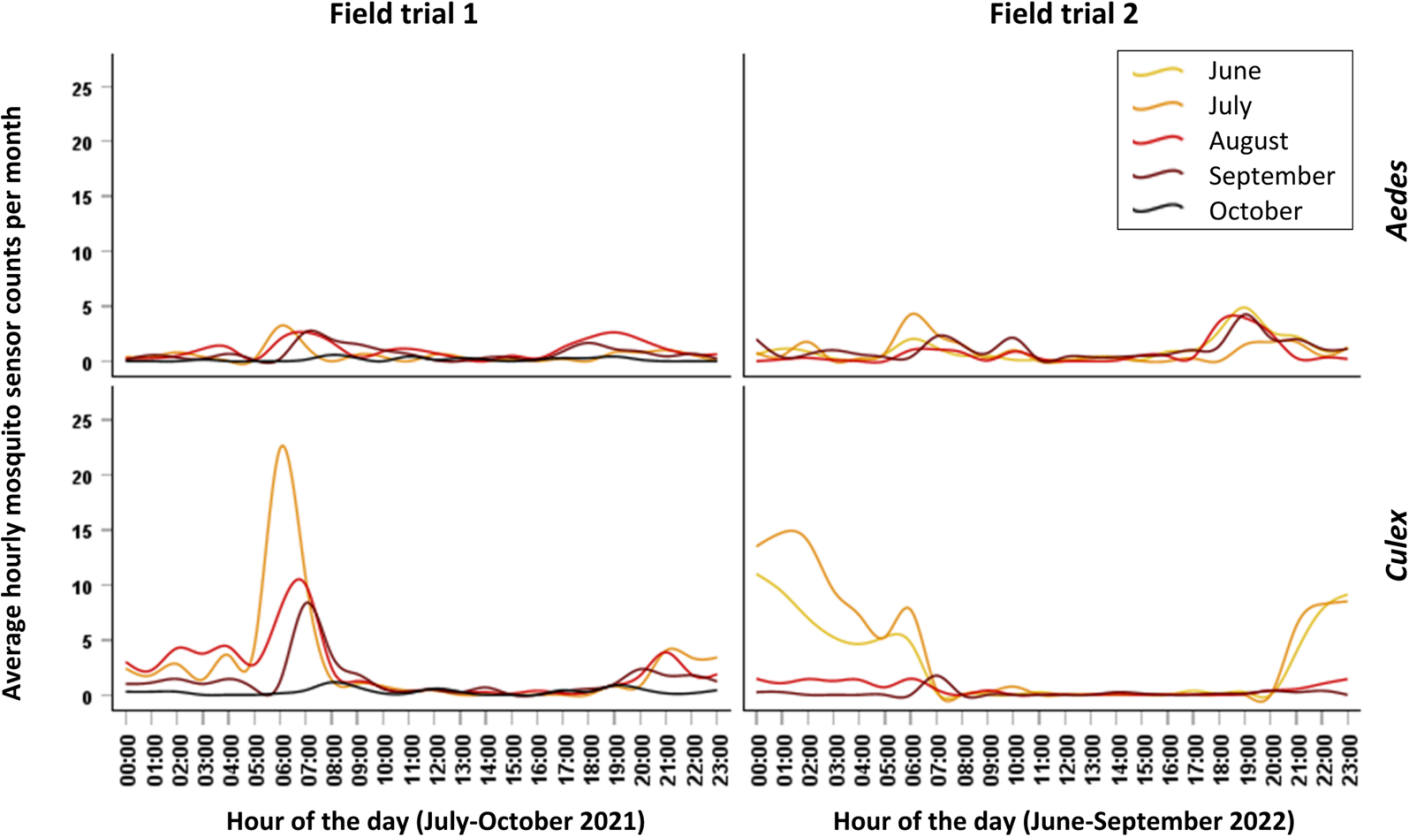
Time series plots of average hourly sensor count per month for the target mosquito genera

The daily and monthly/seasonal activities of *Aedes* and *Culex* mosquitoes for each field trial are represented in Fig. 7. In both field trials, the peak hourly counts for *Culex* mosquitoes (*Cx. pipiens*) are higher than those of *Aedes* (mostly *Ae. albopictus* but also *Ae. caspius* during September and October in field trial 1).

There is a noticeable difference in the activity of *Culex* between the two sites. Regarding the daily activity of *Culex*, in field trial 1 there was a high and pronounced peak of activity in the morning, after sunrise, and a lower and less pronounced peak in the evening at sunset. The morning peak was at 06:00–08:00 in July, which shifted to 07:00–08:30 in August, 07:30–09:00 in September, and 08:00–09:30 in October, but with very low counts. The evening peak was at 21:30–23:00 in July and August and at 20:00–21:00 in September. In field trial 2, *Culex* activity was apparent only during the dark photoperiod, starting around 21:00, just before sunset in the first and second months of summer (June and July) and continuing overnight until sunrise at around 07:00 although the overnight counts were much lower in late summer and early Autumn (August and September) than in June and July.

Regarding the daily activity of *Aedes*, there were generally two peaks of activity per day in both field trials: one in the early morning and one in the evening before the sunset. In field trial 1, the morning peak was at 06:00–07:00 in July and 07:30–8:30 in August and September, and the evening peak was at 18:00–21:00 in August and at 19:00 in September. In field trial 2, the morning peak was at 06:00–07:00 in June and July (being more pronounced in July) and at 07:00–08:00 and 10:00–11:00 in September, with an evening peak between 18:00 and 20:00 from June to September.

## Discussion

The present work tested the performance of an automated mosquito classification system in the field. The system comprises a commercial mosquito suction trap, optical sensor, and ML pipeline, enabling target mosquitoes (*Aedes* and *Culex*) to be discriminated from other insects which entered the trap and the genus and sex of these target mosquitoes to be classified. The data provided by the system also enable the real-time dynamics of the target mosquito populations to be determined with a time resolution as fine as 1 s. The ML model was trained using recordings from thousands of mosquitoes raised in the laboratory which flew through the sensor under different ambient temperature regimes.

The system distinguished the designated target mosquitoes (*Aedes* and *Culex*) from other flying insects that entered the trap with an average BA of 95.5% for the two field trials combined, meaning that the total number of target mosquitoes counted by the sensor was very similar to the number of mosquitoes counted manually. This suggests that the system would be suitable for mosquito surveillance and control activities such as: (i) identifying the start and end of a mosquito activity season, (ii) monitoring seasonal tendencies to prioritize geographical areas of intervention, or (iii) doing quality control checks of control measures aimed at reducing mosquito populations.

The BA results for mosquito target detection were not correlated with the proportion of target mosquitoes in the catch. This is in contrast to the results presented in Day et al. [23] where the mean daily accuracy of the BG-Counter sensor ranged from 9.4 to 80.1% across sites and was highly dependent on the proportion of mosquitoes in the catch, giving high levels of accuracy (80.1%) only in one site when the mean daily proportion of mosquitoes was high (89%).In our work, the overall proportion of target mosquitoes was 39.8%, and the BA for target mosquito detection was high (from 92.9 to 100%) for all collection cycles, even with a proportion of target mosquitoes as low as 3%. These results indicate that the present system performs target mosquito detection with a low rate of false positives. This result can be advantageous in routine mosquito surveillance programs in which carbon dioxide is usually substituted by a more cost-effective attractant such as a chemical lure, which leads to a lower proportion of mosquitoes in the catch [31].

In this work, we also described the automated classification of target mosquitoes by genus and sex in the field, which represents an advance in the state of the art [13–17, 23, 24]. The system classified *Aedes* females, *Aedes* males, *Culex* females, and *Culex* males with an average BA of 88.8%. This can be very useful for public health agencies and biological research in order to detect possible introductions of *Aedes* invasive mosquitoes in new areas where autochthonous *Aedes* species are not present or monitor population dynamics of *Aedes* and *Culex* mosquitoes. The genus and sex classification may also be useful as an indicator of vectorial capacity for arboviruses in urban and peri-urban areas where urbanization processes have a major impact on species richness and favor the spread of invasive anthropophilic vectors such as *Ae. albopictus* [32–34]. The fact that the BA for genus and sex in the field (88.8%) was only slightly below the result obtained in the laboratory (93.9%) indicates that the ML model, which was developed under controlled laboratory conditions, has generalized well to mosquitoes in the field and validates the methodology developed to train the model.

The daily activity patterns of *Aedes* and *Culex* mosquitoes were monitored in this study taking advantage of the high time resolution of the surveillance system. A bimodal activity coinciding with the daylight hours was identified for *Aedes* mosquitoes in both field trials as previously reported for this species [24]. *Culex* exhibit a typical endogenous night activity in field trial 2 but showed an unexpected peak just after sunrise in field trial 1. This plasticity in behavior could be explained by factors such as host availability, environmental conditions, predator inactivity, or bioform type [35]. Moreover, seasonality can have a considerable impact on vector feeding preferences which may drive the transmission of zoonotic pathogens to humans, amplifying the scope of an epidemic [36]. In our study, we observed a progressive shift of the early morning activity peak of *Aedes* and *Culex* to later hours over the duration of each trial from early summer to early autumn coinciding with the sunrise. Temporal variations in the activity of mosquito vectors can have a considerable impact on pathogen transmission [37], so a proper characterization of daily dynamics of local vector species may be of value for risk assessment and control programs [38].

Overall, the system presented here provides several advantages with respect to conventional manual surveillance methods: (i) it significantly reduces the manual effort to gather and inspect each catch bag, especially when target mosquitoes must be sorted from a large number of non-target insects, and to manually record the results; (ii) it provides classification results much earlier than what is possible in routine monitoring programs with collection cycles of 7 to 15 days, enabling a faster epidemiological response when needed; (iii) it is not subject to the effects of predation and degradation of the sample; (iv) it associates a time of capture stamp to each classification result, enabling the activity dynamics of the target insects to be determined with time resolutions down to one second; (v) the server provides automated results in the form of tables and graphs which may be downloaded or visualized on the server itself and may feed risks maps via the application programming interface. The system also provides the following advantages compared to alternative automated mosquito classification systems [13–17, 23, 24]: (i) it provides classification of target mosquito genus and sex in the field, which has not been reported in the scientific literature to date; (ii) it provides reasonably good classification accuracy results (88.8%) for the target *Aedes* and *Culex* mosquito species over the range of ambient temperatures in which these species are known to be active, independently of the proportion of mosquitoes in the catch; (iii) it may be used with existing commercial mosquito traps used in routine entomological surveillance, allowing manual collection and inspection if needed. We believe that the good performance of the system presented is due to the combination of a sensing zone of significant volume, with a dataset that is closer to field conditions, resulting in a more efficient ML model for mosquito classification.

The technology described in this work has been shown to be a valuable tool in urban and peri-urban settings to monitor *Ae. albopictus* and *Cx. pipiens* populations, two of the major vector species in temperate areas [2], and has further potential. However, further work should be done to fully validate the findings by performing more field trials, especially in rural settings, with these and other mosquito species, including autochthonous *Aedes* and *Culex* species for which the model has not been trained. It would also be interesting to perform a more extensive training with additional biological traits (e.g. include different developmental stages of female’s gonotrophic cycle). Furthermore, it should be noted that, with the exception of urban and peri-urban areas dominantly colonized by *Ae. albopictus*, genus identification may be insufficient in some regions for informing about vectorial capacity for arboviruses. Consequently, to enable the system to be applied in a broader range of geographical regions, it would be desirable to achieve species identification by training the sensor with other vector species of medical importance such as *Aedes aegypti, Culex quinquefasciatus*, or the *Anopheles gambiae* complex.

## Conclusions

This work describes the field evaluation of an optical sensor which operates with commercial mosquito traps routinely used in entomological surveillance. This approach enables the sensor to be integrated into conventional mosquito surveillance methods to provide automatic, high temporal resolution monitoring of populations of *Aedes* and *Culex* mosquitoes, two of the most concerning genera in terms of arbovirus transmission. The system automatically discriminates these target mosquitoes from non-target insects in the catch and classifies the target mosquitoes according to genus and sex, which overcomes the manual effort associated with conventional methods to periodically visit the trap and to manually classify the contents of the catch. The system evaluated in the field in the present work, therefore, represents a significant improvement in the state of the art of mosquito surveillance.

### Supplementary Information

**Additional file 1: Table S1.** Confusion matrix showing the results of the ML model for genus and sex classification in laboratory conditions.

**Additional file 2: Table S2. **Manual counts and sensors counts for target mosquito detection and genus and sex classification, presented per collection cycle for both field trials.

## Data Availability

The datasets used and/or analyzed during the current study are available from the corresponding author on reasonable request.

## Abbreviations

BA: Balanced accuracy
CHIKV: Chikungunya virus
DENV: Dengue virus
FN: False negatives
FP: False positives
MBD: Mosquito-borne disease
ML: Machine learning
Se: Sensitivity
Sp: Specificity
TN: True negatives
TP: True positives
WNV: West Nile virus
YFV: Yellow fever virus
ZIKV: Zika virus
