# Supplementary material for: Field evaluation of an automated mosquito surveillance system which classifies Aedes and Culex mosquitoes by genus and sex
Source: Parasit Vectors. 2024 Mar 1;17:97. doi: 10.1186/s13071-024-06177-w (PMC10905882; doi:10.1186/s13071-024-06177-w)
Supplement: Supplementary file 1 — Additional file 1: Table S1. Confusion matrix showing the results of the ML model for genus and sex classification in laboratory conditions. [file 13071_2024_6177_MOESM1_ESM.docx]

| **Predicted** | ***Aedes* female** | ***Aedes* male** | ***Culex* female** | ***Culex* male** |
| --- | --- | --- | --- | --- |
| **Actual** |  |  |  |  |
| ***Aedes* female** | 206 | 4 | 21 | 9 |
| ***Aedes* male** | 8 | 225 | 2 | 21 |
| ***Culex* female** | 8 | 1 | 243 | 0 |
| ***Culex* male** | 13 | 5 | 0 | 234 |
