# Supplementary material for: Field evaluation of an automated mosquito surveillance system which classifies Aedes and Culex mosquitoes by genus and sex
Source: Parasit Vectors. 2024 Mar 1;17:97. doi: 10.1186/s13071-024-06177-w (PMC10905882; doi:10.1186/s13071-024-06177-w)
Supplement: Supplementary file 2 — Additional file 2: Table S2. Manual counts and sensors counts for target mosquito detection and genus and sex classification, presented per collection cycle for both field trials. [file 13071_2024_6177_MOESM2_ESM.docx]

| **Field  trial** | **Date** | | **Manual counts** | | | | | | **Sensor counts** | | | | |
| --- | --- | --- | --- | --- | --- | --- | --- | --- | --- | --- | --- | --- | --- |
|  | **Start** | **End** | **Non-target insects** | **Target mosquitoes** | ***Culex*  female** | ***Culex*  male** | ***Aedes*  female** | ***Aedes* male** | **Target mosquitoes** | ***Culex*  female** | ***Culex*  male** | ***Aedes*  female** | ***Aedes*  male** |
| 1 | 30/06/2021 | 01/07/2021 | 145 | 95 | 90 | 2 | 3 | 0 | 100 | 88 | 1 | 9 | 2 |
| 1 | 07/07/2021 | 08/07/2021 | 108 | 88 | 80 | 2 | 5 | 1 | 94 | 72 | 2 | 19 | 1 |
| 1 | 08/07/2021 | 09/07/2021 | 70 | 28 | 24 | 1 | 1 | 2 | 35 | 27 | 1 | 6 | 1 |
| 1 | 28/07/2021 | 29/07/2021 | 124 | 66 | 54 | 7 | 5 | 0 | 69 | 56 | 6 | 7 | 0 |
| 1 | 29/07/2021 | 30/07/2021 | 89 | 84 | 77 | 4 | 2 | 1 | 93 | 76 | 0 | 14 | 3 |
| 1 | 03/08/2021 | 04/08/2021 | 104 | 117 | 112 | 1 | 2 | 2 | 111 | 89 | 0 | 19 | 3 |
| 1 | 04/08/2021 | 05/08/2021 | 70 | 41 | 35 | 4 | 1 | 1 | 45 | 33 | 1 | 8 | 3 |
| 1 | 10/08/2021 | 11/08/2021 | 168 | 91 | 56 | 1 | 24 | 10 | 98 | 62 | 1 | 26 | 9 |
| 1 | 11/08/2021 | 12/08/2021 | 187 | 79 | 47 | 5 | 14 | 13 | 87 | 48 | 6 | 22 | 11 |
| 1 | 17/08/2021 | 18/08/2021 | 111 | 99 | 67 | 0 | 20 | 12 | 101 | 62 | 2 | 27 | 10 |
| 1 | 18/08/2021 | 19/08/2021 | 143 | 81 | 62 | 5 | 9 | 5 | 83 | 59 | 2 | 15 | 7 |
| 1 | 24/08/2021 | 25/08/2021 | 150 | 87 | 66 | 3 | 10 | 8 | 70 | 51 | 3 | 9 | 7 |
| 1 | 25/08/2021 | 26/08/2021 | 127 | 47 | 30 | 2 | 12 | 3 | 39 | 19 | 1 | 13 | 6 |
| 1 | 31/08/2021 | 01/09/2021 | 258 | 72 | 62 | 0 | 5 | 5 | 67 | 41 | 0 | 22 | 4 |
| 1 | 01/09/2021 | 02/09/2021 | 169 | 59 | 49 | 1 | 9 | 0 | 58 | 32 | 0 | 22 | 4 |
| 1 | 07/09/2021 | 08/09/2021 | 180 | 69 | 56 | 1 | 10 | 2 | 62 | 45 | 0 | 15 | 2 |
| 1 | 08/09/2021 | 09/09/2021 | 143 | 88 | 69 | 0 | 15 | 4 | 82 | 54 | 1 | 26 | 1 |
| 1 | 14/09/2021 | 15/09/2021 | 158 | 51 | 30 | 3 | 15 | 3 | 44 | 27 | 3 | 13 | 1 |
| 1 | 15/09/2021 | 16/09/2021 | 150 | 23 | 16 | 5 | 1 | 1 | 24 | 16 | 1 | 4 | 3 |
| 1 | 21/09/2021 | 22/09/2021 | 211 | 60 | 37 | 2 | 21 | 0 | 51 | 32 | 1 | 18 | 0 |
| 1 | 28/09/2021 | 29/09/2021 | 97 | 36 | 26 | 1 | 9 | 0 | 32 | 23 | 1 | 8 | 0 |
| 1 | 29/09/2021 | 30/09/2021 | 149 | 36 | 20 | 0 | 16 | 0 | 29 | 20 | 0 | 7 | 2 |
| 1 | 05/10/2021 | 06/10/2021 | 295 | 18 | 8 | 2 | 8 | 0 | 18 | 12 | 1 | 5 | 0 |
| 1 | 06/10/2021 | 07/10/2021 | 199 | 10 | 5 | 1 | 4 | 0 | 8 | 6 | 0 | 0 | 2 |
| 1 | 13/10/2021 | 14/10/2021 | 263 | 8 | 2 | 1 | 5 | 0 | 8 | 6 | 0 | 1 | 1 |
| 1 | 14/10/2021 | 15/10/2021 | 234 | 7 | 3 | 0 | 4 | 0 | 6 | 4 | 0 | 2 | 0 |
| 1 | 19/10/2021 | 20/10/2021 | 178 | 17 | 10 | 3 | 4 | 0 | 22 | 14 | 0 | 7 | 1 |
| 1 | 20/10/2021 | 21/10/2021 | 168 | 5 | 4 | 1 | 0 | 0 | 7 | 3 | 1 | 2 | 1 |
| 1 | 26/10/2021 | 27/10/2021 | 50 | 8 | 5 | 1 | 2 | 0 | 6 | 3 | 1 | 2 | 0 |
| 2 | 01/06/2022 | 03/06/2022 | 273 | 29 | 9 | 0 | 16 | 4 | 40 | 13 | 1 | 15 | 11 |
| 2 | 08/06/2022 | 12/06/2022 | 291 | 104 | 21 | 4 | 44 | 35 | 103 | 23 | 3 | 41 | 36 |
| 2 | 15/06/2022 | 17/06/2022 | 210 | 460 | 395 | 19 | 34 | 12 | 431 | 350 | 8 | 52 | 21 |
| 2 | 21/06/2022 | 23/06/2022 | 128 | 259 | 231 | 8 | 18 | 2 | 200 | 156 | 3 | 31 | 10 |
| 2 | 13/07/2022 | 15/07/2022 | 116 | 377 | 330 | 19 | 21 | 7 | 368 | 300 | 9 | 38 | 21 |
| 2 | 25/07/2022 | 27/07/2022 | 133 | 130 | 105 | 7 | 15 | 3 | 119 | 88 | 3 | 18 | 10 |
| 2 | 03/08/2022 | 05/08/2022 | 124 | 74 | 55 | 6 | 6 | 7 | 73 | 51 | 9 | 7 | 6 |
| 2 | 10/08/2022 | 12/08/2022 | 181 | 76 | 22 | 6 | 32 | 16 | 64 | 22 | 3 | 27 | 12 |
| 2 | 16/08/2022 | 19/08/2022 | 78 | 77 | 37 | 2 | 30 | 8 | 70 | 31 | 2 | 22 | 15 |
| 2 | 22/08/2022 | 23/08/2022 | 62 | 58 | 13 | 4 | 29 | 12 | 46 | 11 | 1 | 20 | 14 |
| 2 | 24/08/2022 | 25/08/2022 | 27 | 17 | 2 | 0 | 7 | 8 | 14 | 0 | 1 | 6 | 7 |
| 2 | 30/08/2022 | 01/09/2022 | 151 | 75 | 21 | 7 | 26 | 21 | 68 | 12 | 7 | 27 | 22 |
| 2 | 07/09/2022 | 08/09/2022 | 144 | 45 | 3 | 2 | 24 | 16 | 52 | 1 | 2 | 29 | 20 |
| 2 | 13/09/2022 | 15/09/2022 | 80 | 72 | 11 | 2 | 43 | 16 | 95 | 17 | 3 | 52 | 23 |
| 2 | 19/09/2022 | 21/09/2022 | 166 | 77 | 40 | 6 | 20 | 11 | 83 | 46 | 4 | 15 | 18 |
